# Amino­silanes derived from 1*H*-benzimidazole-2(3*H*)-thione

**DOI:** 10.1107/S2053229615014503

**Published:** 2015-08-12

**Authors:** Juliana Palomo-Molina, Efrén V. García-Báez, Rosalinda Contreras, Kayim Pineda-Urbina, Angel Ramos-Organillo

**Affiliations:** a Facultad de Ciencias Químicas, Universidad de Colima, Carretera Coquimatlán-Colima, Coquimatlán Colima 28400, Mexico; b Unidad Profesional Interdisciplinaria de Biotecnología, Instituto Politécnico Nacional, Avenida Acueducto s/n, Barrio La Laguna Ticomán, México DF 07340, Mexico; c Departamento de Química, Centro de Investigación y de Estudios Avanzados del IPN, Apartado Postal 14-740, México DF 07000, Mexico; d Barrio La Laguna Ticomán, México DF 07340, Mexico

**Keywords:** 1*H*-benzimidazole-2(3*H*)-thione, amino­silanes, crystal structure, N—H⋯S inter­actions, hydrogen bonding

## Abstract

Two new mol­ecular structures, namely 1,3-bis­(tri­methyl­silyl)-1*H*-benzimidazole-2(3*H*)-thione, C_13_H_22_N_2_SSi_2_, (2), and 1-tri­methyl­silyl-1*H*-benzimidazole-2(3*H*)-thione, C_10_H_14_N_2_SSi, (3), are reported. Both systems were derived from 1*H*-benzimidazole-2(3*H*)-thione. Noncovalent C—H⋯π inter­actions between the centroid of the benzmidazole system and the SiMe_3_ groups form helicoidal arrangements in (2). Dimerization of (3) results in the formation of *R*_2_^2^(8) rings *via* N—H⋯S inter­actions, along with parallel π–π inter­actions between imidazole and benzene rings.

## Abstract 2

In two tri­methyl­silyl-substituted 1*H*-benzimidazole-2(3*H*)-thiones, noncovalent C—H⋯π inter­actions between the centroid of the benzmidazole system and the SiMe_3_ groups form helicoidal arrangements in one, and dimerization results in the formation of 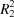
(8) rings *via* N—H⋯S inter­actions, along with parallel π–π inter­actions between imidazole and benzene rings, in the second compound.

## Introduction   

1*H*-Benzimidazole-2(3*H*)-thione, (1) (see Scheme 1), is a planar mol­ecule with two substitutable acidic H atoms. The N atoms of this mol­ecule have demonstrated the ability to form Lewis acid–base coordination compounds. Under basic conditions, the corresponding salt of (1) has been shown to react with *p*-block elements (O’Sullivan & Wallis, 1972 ▸).
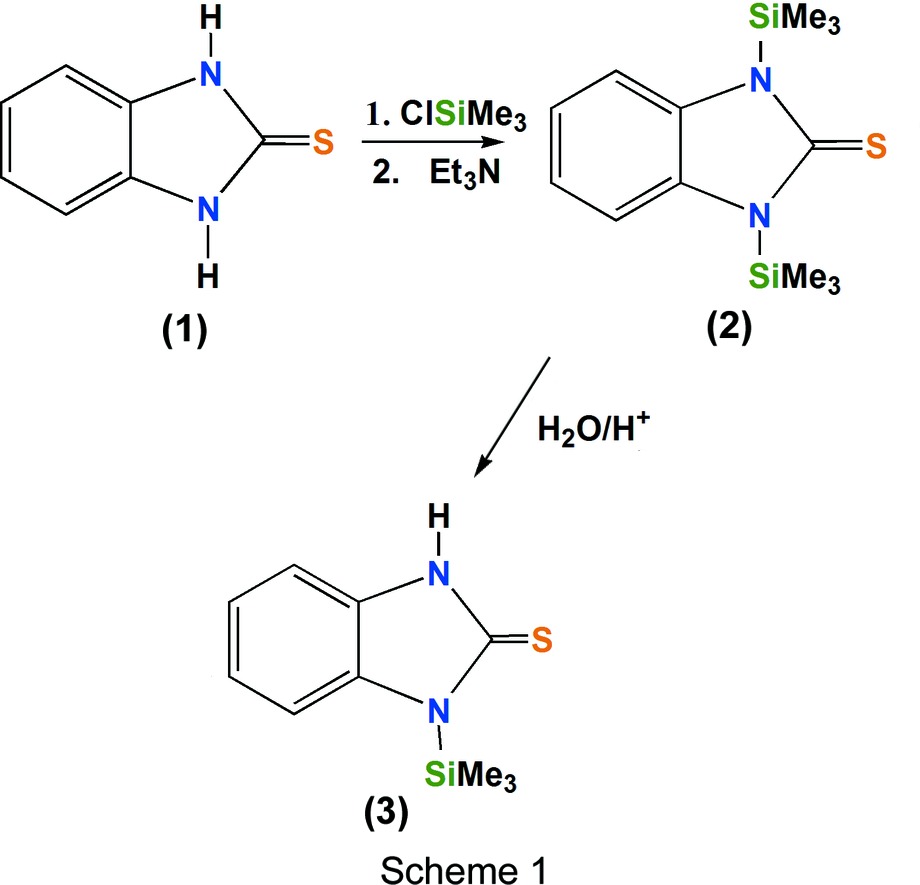


The 1*H*-benzimidazole-2(3*H*)-thione heterocycle has been found in compounds with biological activity, such as progesterone agonists (Zhang *et al.*, 2007 ▸). Anti­nematode activity was evaluated for {[(1*H*-benzimidazol-2-yl)thio]­acetyl}­piperazine (Mavrova *et al.*, 2010 ▸), while 2-(alkyl­thio)­benzimidazole with a β-lactam ring pre­sented anti­bacterial and anti­fungal activities (Desai & Desai, 2006 ▸). Isomeric 2-(methylthio)­benzimidazole compounds were synthesized as acyclic analogues of the HIV-1 RT inhibitor ring system (Gardiner & Loyns, 1995 ▸). More recently, isoxazole–mer­capto­benzimid­azole hybrids have presented analgesic and anti-inflammatory activities (Shravankumar *et al.*, 2013 ▸). Furthermore, a wide range of biological activities have been reported for the benzimid­azole fragment, such as anti­fungal, anti­bacterial, vasodilator, antispasmodic, anti-ulcer (Akkurt *et al.*, 2012 ▸), anti­microbial (De Almeida *et al.*, 2007 ▸), anti­histamine (Mor *et al.*, 2004 ▸), neutropic (Bakhareva *et al.*, 1996 ▸) and analgesic (Anandarajagopal *et al.*, 2010 ▸). Additionally, alkyl­silyl-substituted benzimidazole has shown *in vitro* cytotoxicity, for example, 1-[3-(tri­methyl­silyl)propyl]benz­imid­azole inhibits carcinoma S180 tumour (Lukevics *et al.*, 2001 ▸). In 2012, 1-{[dimethyl(phenyl)silyl]methyl}-3-(2-phenyl­ethyl)-1-benzimidazol-3-ium bromide monohydrate was synthesized and its crystal structure elucidated (Akkurt *et al.*, 2012 ▸). Silylated compounds are stable at low temperatures and, in some cases, under atmospheric conditions. Amino­silanes are soluble in nonpolar solvents, while the presence of tri­methyl­silyl groups increases the volatility of the organic fragments, most of which can be distilled without decomposition and, sometimes, even crystallized (Ghose & Gilchrist, 1991 ▸). Alk­oxy­silanes, thio­silanes and amino­silanes are stable at low temperatures, while the last class become unstable under atmospheric conditions (Colvin, 1981 ▸).

We report here the crystal structures of two new tri­methyl­silyl-substituted derivatives of 1*H*-benzimidazole-2(3*H*)-thione, namely 1,3-bis­(tri­methyl­silyl)-1*H*-benzimid­azole-2(3*H*)-thione, (2), and 1-tri­methyl­silyl-1*H*-benzimid­azole-2(3*H*)-thione, (3).

## Experimental   

All reagents were purchased from Aldrich and were used as received. All solvents were dried before use. ^1^H NMR (300.13185 MHz) and ^13^C NMR (75.47564 MHz) analyses in CDCl_3_ were performed on a Bruker 300 MHz spectrometer, using TMS as the inter­nal reference. Chemical shifts (δ) are reported in p.p.m. IR spectra were recorded on a Perkin–Elmer FT–IR 1600 spectrophotometer in the 4000–400 cm^−1^ range. Elemental analyses were performed in a Thermofinniga Flash 112 instrument under standard conditions.

### Synthesis and crystallization   

Compound (2) was obtained by mixing 1*H*-benzimidazole-2(3*H*)-thione (0.5 g, 3.3 mmol) and chloro­tri­methyl­silane (0.89 ml, 75.9 mg, 6.9 mmol) in tri­ethyl­amine (15 ml). The reaction was kept under constant stirring and reflux for 6 h. The resulting compound was a yellow liquid (yield 92%, 1.87 g) which solidified after 24 h. Crystals of (2) suitable for X-ray diffraction analysis were collected. MS: *m*/*z* (intensity, %): 294 (*M* ^+^, 100), 206 (25), 150 (11); IR (KBr, ν_max_, cm^−1^): 1623 (C=N), 1514 and 1470 (N—C—S), 1181 (Si—N), 714 and 710 (Si—C); ^1^H NMR (C_6_D_6_/THF, 1:1): δ *AA*′*BB*′ 7.26 (*m*, H4, H7), 7.04 (*m*, H5, H6), 0.73 (*s*, H_Me_); ^13^C NMR: δ 182.3 (C2), 112.2 (C4, C7), 122.6 (C5, C6), 2.5 (C_Me_). Elemental analysis calculated for C_13_H_22_N_2_SSi_2_: C 53.01, H 7.53, N 9.51, S 10.89%; found: C 53.03, H 7.60, N 9.60, S 10.69%.

Compound (3) was obtained from the partial hydrolysis of (2); both (2) and (3) are readily hydrolysed under atmospheric conditions. This compound was not analysed by spectroscopic techniques. However, crystals of (3) suitable for X-ray diffraction analysis were obtained from a hexane solution and a single crystal immersed in oil was analysed.

### Refinement   

Crystal data, data collection and structure refinement details are summarized in Table 1 ▸. H atoms were included in geometrically calculated positions, riding on the C or N atoms to which they were bonded. C—H distances were restrained to 0.93 (aromatic) or 0.96 Å (methyl) and the N—H bond length was restrained to 0.86 Å. H-atom displacement parameters were set at *U*_iso_(H) = 1.5*U*_eq_(C) for methyl H atoms and at 1.2*U*_eq_(C,N) otherwise.

## Results and discussion   

Compound (2) crystallizes in the ortho­rhom­bic space group *P*2_1_2_1_2_1_. The average N1—Si1—Me_10,11,12_ angle is 109.0 (2)° and the average N1—Si1—Me_13,14,15_ angle is 109.1 (2)°. The Si—N distances of 1.809 (3) and 1.803 (3) Å are slightly longer than those reported previously for 1,3-bis­(tri­methyl­silyl)imidazolidin-2-one [1.739 (7) Å] and 4-methyl-1,3-bis­(tri­methyl­silyl)imidazolidin-2-one [1.745 (3) Å] (Szalay *et al.*, 2005 ▸), which might be caused by the difference in electronegativities of the O and S atoms.

Compound (3) crystallizes with two independent mol­ecules, *A* and *B*, in the asymmetric unit in the monoclinic space group *P*2_1_/*c*. The average N1—Si1—Me_20,21,22_ angle is 108.49 (12)° and the average N11—Si2—Me_23,24,25_ angle is 108.66 (12)°. The Si—N distances are 1.817 (2) and 1.804 (2) Å.

**Figure 1:**
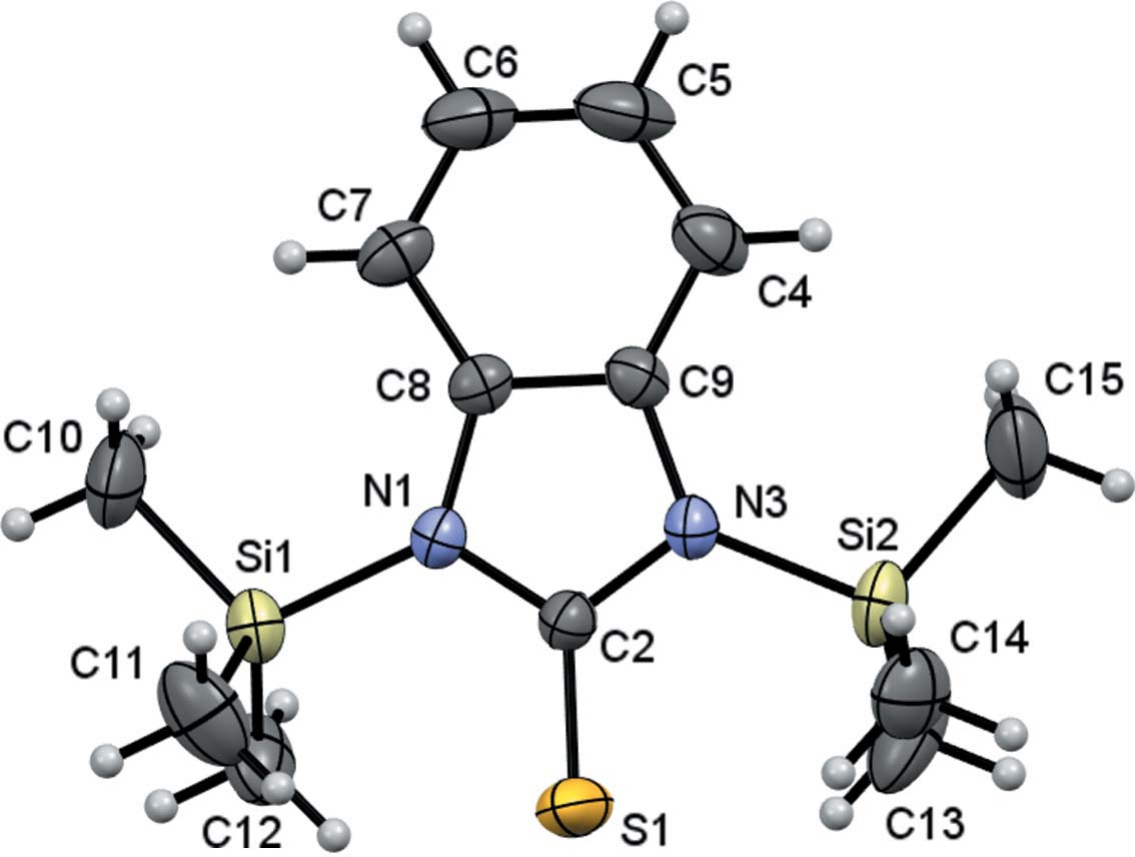
The mol­ecular structure of compound (2), showing the atom-numbering scheme. Displacement ellipsoids are drawn at the 30% probability level.

Overall, compounds (2) and (3) have very similar structures, which are shown in Figs. 1 ▸ and 2 ▸, respectively. Selected bond lengths and angles are listed in Tables 2 ▸ and 3 ▸, respectively. The average C—Si bond length for both compounds is 1.847 (3) Å and the average C—Si—C angle is 109.5 (2)°, in agreement with *sp* ^3^-hybridization of the Si atoms. These values agree with those in similar structures reported previously (Wagler *et al.*, 2010 ▸).

The C=S distances for compounds (2) and (3) range from 1.669 (4) to 1.675 (2) Å. The average N_1,3_—C2=S1 angle is 125.0 (3)° for (2) and the average N_1,11_—C_2,12_=S12 angle is 126.9 (18)° for (3). These angles agree with *sp* ^2^-hybridization of the C and S atoms which is typical of thio­urea groups (Wagler *et al.*, 2010 ▸). The S atom of (3) has a slight displacement of 0.007 (1) Å from the benzimidazole mol­ecular plane, whereas in (2), the S atom is out of the plane by 0.155 (2) Å. This displacement could be caused by noncovalent intra­molecular inter­actions between the S-atom nucleus and both Si atoms, or between the methyl H atoms and the S atom. Compound (2) presents four noncovalent C—H⋯S inter­actions (Table 4 ▸), with C⋯S distances ranging from 2.77 to 2.96 Å and angles ranging from 122 to 125°, which amount to less than the sum of the van der Waals radii of S and H atoms (3.25 Å; Bondi, 1964 ▸).

Another noncovalent intra­molecular inter­action (Table 5 ▸) was observed in (3), *viz.* C21—H21⋯S1, with a C⋯S distance of 2.83 Å and an angle of 126°, similar to that observed in (2).

Comparing the structures of (2) and (3), it becomes obvious that the fused rings in (2) are not completely flat. Specifically, the thio­urea unit composed of atoms N1/C2/N3/S1 is offset from the mol­ecular plane defined by the benzene ring. This is a consequence of the intra­molecular noncovalent C—H⋯S inter­actions present in the system.

**Figure 3:**
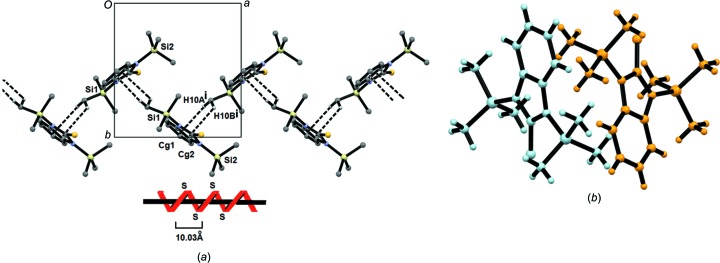
(*a*) The spiral arrangement for (2) and (*b*) the overlap of the helix along the direction of the *a* axis.

Fig. 3 ▸(*a*) shows the spiral arrangement of (2), which forms a linking inter­action between mol­ecules through the imidazole ring (C10—H10*A*⋯*Cg*1 = 2.94 Å; *Cg*1 is the centroid of the imidazole ring) and the benzene ring [C10—H10*B*⋯*Cg*2 = 2.83 Å; *Cg*2 is the centroid of the benzene ring at (*x* − 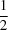, −*y* + 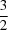, −*z*)]. These inter­actions form a helicoidal repeat unit of 10.03 Å, which extends along the crystallographic *a* axis. Fig. 3 ▸(*b*) presents the helix overlap of this system. A third inter­action, *viz.* C13—H13⋯π(*x* + 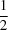, −*y* + 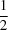, −*z*), has a C⋯π distance of 2.77 Å, which further supports the helicoidal arrangement.

**Figure 4:**
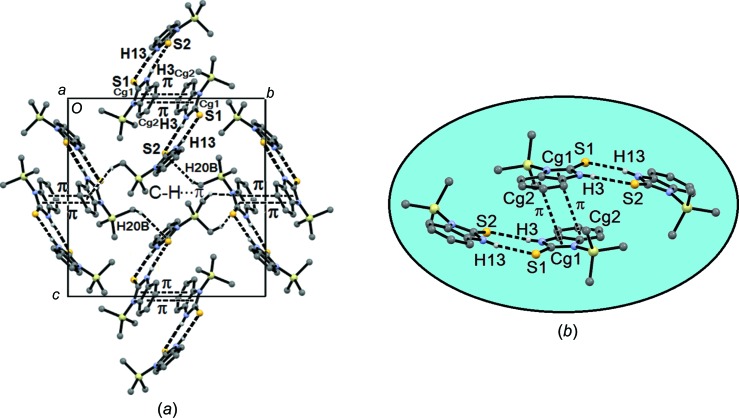
(*a*) The crystal packing diagram of (3) along the direction of the *ab* plane. (*b*) A detailed view of the formation of the 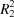
(8) hydrogen-bonding motif and the π–π stacking inter­actions. [Where is the origin in part (*a*)?]

Mol­ecules *A* and *B* of (3) are auto-assembled by N—H⋯S inter­actions (N3—H3⋯S2^i^ = 2.52 Å and N13—H13⋯S1^i^ = 2.45 Å; see Table 5 ▸ for symmetry code). This arrangement forms a cyclic system with an 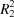
(8) hydrogen-bonding pattern (Bernstein *et al.*, 1995 ▸) (Fig. 4 ▸). Furthermore, π–π inter­actions between the imidazole and benzene rings are observed in the dimerization of the compound and extend in the *ab* plane (Fig. 4 ▸). The distance between the ring centroids in these inter­actions is 3.64 Å (symmetry code: −*x* + 1, −*y* + 1, −*z*). There is an additional inter­molecular C20—H20*B*⋯π(imidazole ring) inter­action of 3.03 Å (symmetry code: −*x* + 1, *y* + 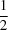, −*z* + 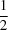) which strengthens the crystalline arrange­ment of (3).

As can be seen, the structures of (2) and (3) have similar parameters around the silyl–amine bond, but while (3) is a dimer formed by classical hydrogen bonding, the structure of (2) is a helix supported by nonclassical interactions.

## Supplementary Material

Crystal structure: contains datablock(s) 2, 3, global. DOI: 10.1107/S2053229615014503/fn3201sup1.cif

Structure factors: contains datablock(s) 2. DOI: 10.1107/S2053229615014503/fn32012sup2.hkl

Structure factors: contains datablock(s) 3. DOI: 10.1107/S2053229615014503/fn32013sup3.hkl

Supporting information file. DOI: 10.1107/S2053229615014503/fn32012sup4.cml

Supporting information file. DOI: 10.1107/S2053229615014503/fn32013sup5.cml

CCDC references: 1416509, 1416508

## Figures and Tables

**Figure 2:**
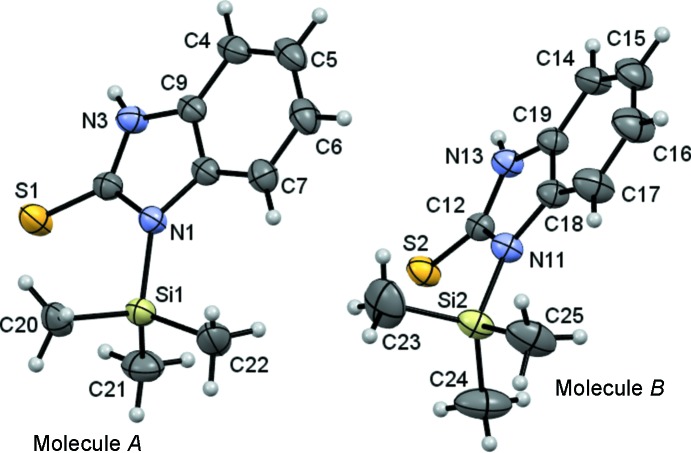
The mol­ecular structures of the two independent molecules of compound (3), showing the atom-numbering schemes. Displacement ellipsoids are drawn at the 30% probability level.

**Table 1:** Experimental details

	(2)	(3)
Crystal data		
Chemical formula	C_13_H_22_N_2_SSi_2_	C_10_H_14_N_2_SSi
*M*_r_	294.56	222.38
Crystal system, space group	Orthorhombic, *P*2_1_2_1_2_1_	Monoclinic, *P*2_1_/*c*
Temperature (K)	293	293
*a*, *b*, *c* ()	10.0302(3), 10.6172(3), 16.2428(6)	9.8057(2), 15.8032(4), 15.8658(5)
, , ()	90, 90, 90	90, 93.859(1), 90
*V* (^3^)	1729.74(10)	2453.01(11)
*Z*	4	8
Radiation type	Mo *K*	Mo *K*
(mm^1^)	0.31	0.33
Crystal size (mm)	0.25 0.20 0.10 0.15 (radius)	0.20 0.20 0.15 0.15 (radius)

Data collection		
Diffractometer	Nonius KappaCCD area-detector diffractometer	Nonius KappaCCD area-detector diffractometer
Absorption correction	Spherical (Dwiggins, 1975 ▸)	Spherical (Dwiggins, 1975 ▸)
*T*_min_, *T*_max_	0.861, 0.862	0.861, 0.862
No. of measured, independent and observed [*I* > 2(*I*)] reflections	15678, 3889, 2472	29355, 5554, 3199
*R*_int_	0.064	0.096
(sin /)_max_ (^1^)	0.648	0.649

Refinement		
*R*[*F* ^2^ > 2(*F* ^2^)], *wR*(*F* ^2^), *S*	0.048, 0.104, 1.01	0.049, 0.138, 1.00
No. of reflections	3889	5554
No. of parameters	164	259
H-atom treatment	H-atom parameters constrained	H-atom parameters constrained
_max_, _min_ (e ^3^)	0.17, 0.20	0.21, 0.24
Absolute structure	Flack *x* parameter determined using 838 quotients, [(*I* ^+^) (*I* )]/[(*I* ^+^) + (*I* )] (Parsons *et al.*, 2013 ▸)	
Absolute structure parameter	0.01(7)	

Computer programs: *COLLECT* (Nonius, 2000 ▸), *DENZO* and *SCALEPACK* (Otwinowski Minor, 1997 ▸), *SHELXS97* (Sheldrick, 2008 ▸), *SHELXL2014* (Sheldrick, 2015 ▸), and *XPMA* in *ZORTEP* (Zsolnai, 1997 ▸).

**Table 2:** Selected geometric parameters (, ) for (2)

Si1N1	1.809(3)	Si2C13	1.839(6)
Si1C11	1.842(5)	Si2C15	1.854(6)
Si1C12	1.842(5)	Si2C14	1.861(5)
Si1C10	1.847(5)	S1C2	1.669(4)
Si2N3	1.803(3)		
			
N1Si1C11	109.0(2)	N3Si2C14	109.3(2)
N1Si1C12	109.53(19)	C13Si2C14	113.7(3)
C11Si1C12	113.9(3)	C15Si2C14	107.7(3)
N1Si1C10	108.4(2)	C2N1Si1	121.7(3)
C11Si1C10	109.4(3)	C8N1Si1	130.9(3)
C12Si1C10	106.4(3)	C2N3Si2	120.8(3)
N3Si2C13	109.4(2)	C9N3Si2	132.3(2)
N3Si2C15	108.5(2)	N1C2S1	125.1(3)
C13Si2C15	108.2(3)	N3C2S1	124.8(3)
			
C11Si1N1C2	70.3(4)	C14Si2N3C9	113.9(4)
C12Si1N1C2	55.0(4)	Si2N3C9C4	4.8(7)
C10Si1N1C2	170.7(3)	Si2N3C9C8	179.1(3)
C11Si1N1C8	113.2(4)	Si1N1C8C7	10.2(6)
C12Si1N1C8	121.5(4)	Si1N1C8C9	174.1(3)
C10Si1N1C8	5.8(4)	Si1N1C2N3	173.6(2)
C13Si2N3C2	59.4(4)	C8N1C2S1	175.3(3)
C15Si2N3C2	177.2(4)	Si1N1C2S1	7.5(5)
C14Si2N3C2	65.7(4)	Si2N3C2N1	177.2(2)
C13Si2N3C9	121.0(4)	C9N3C2S1	175.9(3)
C15Si2N3C9	3.2(4)	Si2N3C2S1	3.9(5)

**Table 3:** Selected geometric parameters (, ) for (3)

S1C2	1.676(3)	S2C12	1.675(2)
Si1N1	1.817(2)	Si2N11	1.804(2)
Si1C22	1.841(3)	Si2C24	1.827(3)
Si1C20	1.846(3)	Si2C23	1.830(4)
Si1C21	1.850(3)	Si2C25	1.841(3)
			
N1Si1C22	108.72(12)	N11Si2C24	111.21(15)
N1Si1C20	107.62(12)	N11Si2C23	105.51(15)
C22Si1C20	109.24(16)	C24Si2C23	113.3(2)
N1Si1C21	109.12(13)	N11Si2C25	109.27(13)
C22Si1C21	108.81(18)	C24Si2C25	106.95(19)
C20Si1C21	113.23(16)	C23Si2C25	110.6(2)
C2N1Si1	122.00(16)	C12N11Si2	123.12(16)
C8N1Si1	130.56(17)	C18N11Si2	128.88(17)
N3C2S1	125.48(19)	N13C12S2	125.02(19)
N1C2S1	126.65(18)	N11C12S2	127.12(18)
			
C22Si1N1C2	176.3(2)	C24Si2N11C12	56.7(3)
C20Si1N1C2	65.5(2)	C23Si2N11C12	66.5(2)
C21Si1N1C2	57.8(2)	C25Si2N11C12	174.5(2)
C22Si1N1C8	1.1(3)	C24Si2N11C18	133.4(3)
C20Si1N1C8	117.1(2)	C23Si2N11C18	103.4(3)
C21Si1N1C8	119.6(2)	C25Si2N11C18	15.6(3)
C9N3C2S1	179.14(18)	C19N13C12S2	179.11(18)
Si1N1C2N3	177.32(16)	Si2N11C12N13	171.28(16)
C8N1C2S1	178.90(19)	C18N11C12S2	179.38(19)
Si1N1C2S1	3.2(3)	Si2N11C12S2	8.8(3)
Si1N1C8C9	177.06(17)	Si2N11C18C17	8.5(5)
Si1N1C8C7	3.0(4)	Si2N11C18C19	171.09(18)

**Table 4:** Hydrogen-bond geometry (, ) for (2)

*D*H*A*	*D*H	H*A*	*D* *A*	*D*H*A*
C11H11*B*S1	0.96	2.96	3.564(7)	122
C12H12*C*S1	0.96	2.77	3.415(5)	125
C13H13*B*S1	0.96	2.79	3.423(7)	125
C14H14*C*S1	0.96	2.86	3.480(5)	123

**Table 5:** Hydrogen-bond geometry (, ) for (3)

*D*H*A*	*D*H	H*A*	*D* *A*	*D*H*A*
N3H3S2^i^	0.86	2.52	3.374(2)	170
N13H13S1^i^	0.86	2.45	3.282(2)	164
C21H21*B*S1	0.96	2.83	3.480(4)	126

Symmetry code: (i) 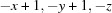.
